# A Novel Marker-Based Registration Method for Simultaneous Preclinical PET/MR with a Non-Stationary PET Detector

**DOI:** 10.21203/rs.3.rs-9974966/v1

**Published:** 2026-07-02

**Authors:** Leo Marecki, Suyog Pol, Pawel Markiewicz, Robert Zivadinov, Ferdinand Schweser

**Affiliations:** 1 Department of Biomedical Engineering, University at Buffalo, State University of New York, Buffalo, NY 14260, USA.; 2 Buffalo Neuroimaging Analysis Center, Department of Neurology, School of Medicine and Biomedical Sciences, University at Buffalo, State University of New York, Buffalo, NY 14203, USA.; 3 Center for Biomedical Imaging, University at Buffalo, State University of New York, Buffalo, NY 14203, USA.; 4 School of Computer Science and Digital Technologies, LSBU, London, UK.; 5 Medical Physics and Biomedical Engineering, Hawkes Institute, UCL, London, UK.; 6 Nuclear Medicine, Royal Free Hospital, London, UK.

## Abstract

**Purpose:**

To develop a novel, fully automated marker-based method for registering PET images from a small, non-stationary, retrofitted preclinical PET detector to simultaneously acquired MRI images.

**Methods:**

We manufactured a nose cone tract with geometric markers from a material visible when imaged with a zero-echo-time MRI sequence. A one-time universal calibration determined the relation between the PET image space and the nose cone tract markers. An experiment-specific calibration was needed to determine the location of the nose cone tract markers in laboratory space, and, hence, in MRI image space. The robustness of the method was evaluated through systematic experiments.

**Results:**

Experiments demonstrated successful registration of the two imaging modalities with a spatial registration error below the PET voxel size.

**Conclusion:**

The proposed registration approach is a practical method for registering simultaneously acquired PET and MR images with sub-voxel precision when the PET device is non-stationary and the usable space inside the detector is too small for dual-modality fiducial markers.

## Introduction

Positron emission tomography (PET) is widely used in healthcare and in clinical and preclinical research (1–4). PET has enabled researchers to perform both quantitative and qualitative analyses on cell and tissue functionality through the utilization of radioactive isotopes chemically bound to specific organic molecules (5–8). While PET is a strong method for assessing specific tissue functionality, the lack of endogenous tissue contrast often requires the PET images to be registered to other imaging modalities that can provide anatomical contrast that allows locating the radiotracer uptake, like computed tomography (CT) or, more recently, magnetic resonance imaging (MRI) (1, 2). Numerous PET/MR scanner designs have been proposed that enable simultaneous scanning to minimize total experiment time and enable sophisticated hybrid reconstruction approaches (3–6). Among these scanners, several have been designed specifically for preclinical applications in small animals (7).

The registration of the PET and MR images has been achieved through several different approaches: (i) registration based solely on image intensities, (ii) registration based on dual-modality markers, and (iii) registration based on a one-time calibration with a dedicated phantom (2, 8–12). Each of these approaches has distinct shortcomings: registration based on image intensities is only possible if mutual anatomical contrast is present in both imaging modalities, dual-modality markers requires sufficient space within the PET detector to accommodate both the subject and the fiducial markers, and the one-time calibration requires that the PET scanner can either be placed repeatably in the same position within the MR magnet or never has to be removed after the calibration was performed.

The present study was part of a larger aim to retrofit a small non-stationary PET detector to an ultra-high field preclinical MRI scanner. The PET detector was retrofitted to the MR scanner animal cradle positioning system (previously presented (13), which facilitated an efficient experimental workflow but rendered the physical location of the detector dependent on the relatively imprecise (±1 mm axial) laser-based manual position calibration. Besides, experiments have indicated that even without recalibration of the laser-setpoint, the positioning system had an axial positioning error of ±165 μm (unpublished). The positioning of the radio-frequency (RF) coil inside the PET detector left insufficient physical space to place a dual-modality phantom besides the imaging subject in every experiment.

We developed a registration method that enables registering simultaneously acquired PET scans to MR scans without relying on anatomical features, utilizing fiducial markers, or performing dual-modality calibration studies for every experiment.

## Methods

### Imaging Equipment

We used a miniature prototype PET detector ring (SynchroPET Inc.) originally designed for conscious rat brain imaging (RatCAP) (14). The PET detector ring was equipped with an electromagnetic shield to improve MRI compatibility (14, 15), resulting in inner/outer diameters of 44.0/83.1 mm and a weight of 243 g.

For MRI, we used a 200 mm diameter horizontal-bore 9.4 Tesla small-animal scanner (BioSpec 94/20USR, Bruker Biospin). A shielded volume transmit/receive RF coil for mouse imaging was placed within the PET ring (inner/outer diameters of 23.0/44.0 mm, model T12969V3, Bruker). Preliminary applications of this assembly have been presented previously (16–18).

**Figure 1.**
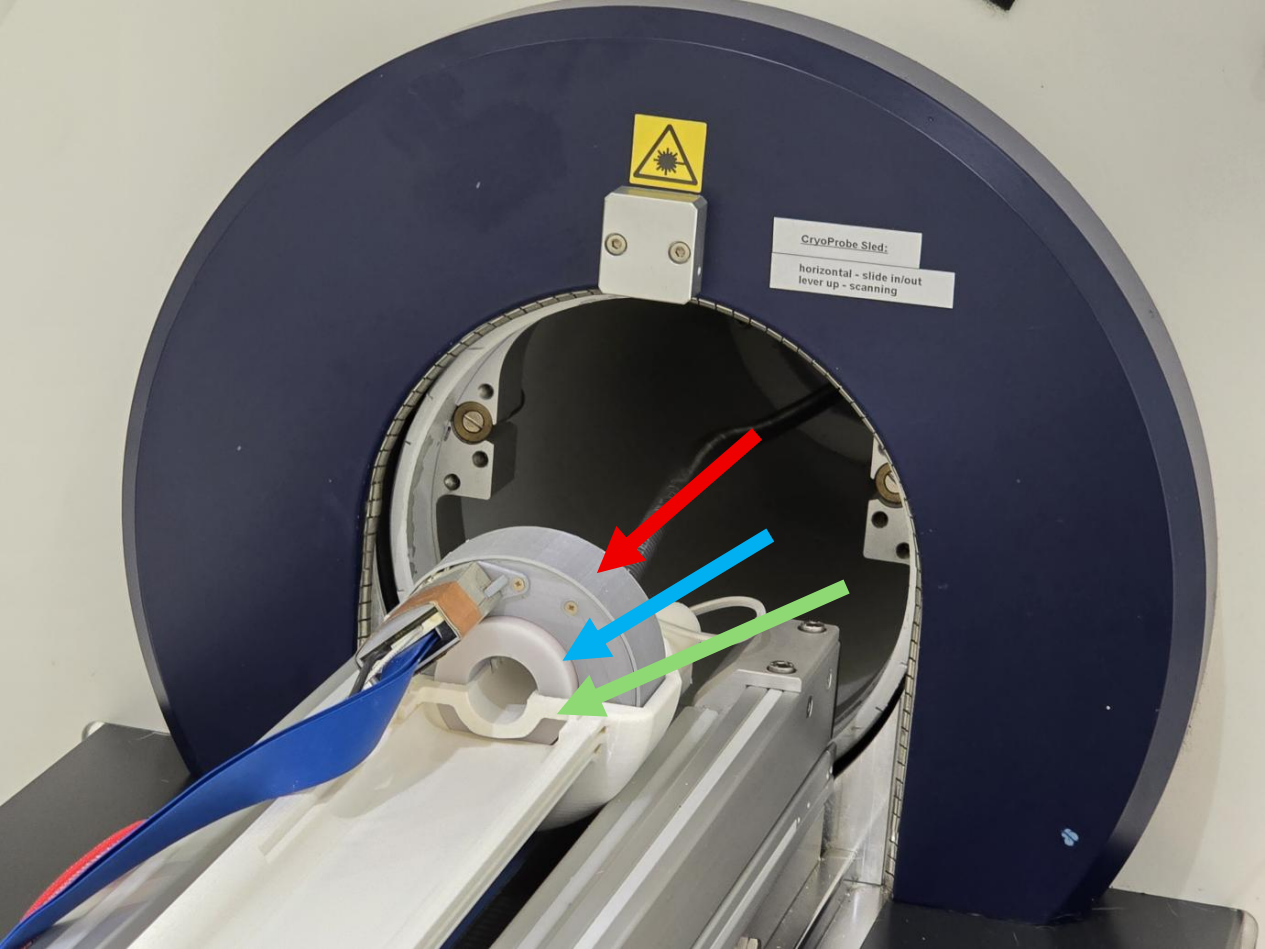
Assembly used in this study. The PET ring (red arrow) is shown in grey, with volume transmit/receive coil in white (blue arrow), and nose cone track (green arrow) placed in custom animal cradle.

We used a custom-designed animal cradle with a dedicated bay for the PET-RF coil assembly that could be attached to the MR scanner motorized subject positioning system (AutoPac, Bruker Biospin). Details of an earlier prototype of this assembly were described elsewhere (19) (manuscript submitted) and are shown in Figure 1.

The MRI system was operated with the vendor provided software (Bruker ParaVision, v6.0.1), and the PET system was operated with a custom prototype software (Matlab, The Mathworks). All PET images were reconstructed with a custom maximum-likelihood expectation-maximization (MLEM) with twenty iterations, initialized using a filtered back-projection (FBP) image with a matrix size of 129 × 129 × 17.0 at 0.25 mm × 0.25 mm × 1.0 mm resolution.

### General approach

The goal of PET and MRI image registration is to transform the PET image coordinate space into the MR image coordinate space.

Due to the limited location reproducibility of the animal positioning system, the affine transform relating the PET image coordinate space to the MR image coordinate space changed with every experimental repositioning of the cradle. This setup prevented using a one-time calibration with a dual-modality phantom to determine the exact physical location of the assembly relative to the magnet isocenter. While a dual modality fiducial marker (visible in both modalities in every experiment) would be a viable component to overcome this shortcoming, the narrow MRI coil inner diameter left little space to accommodate such a marker in typical imaging scenarios. Our approach relied on the fixed spatial relationship between the PET ring and the cradle nose-cone track (NCT).

Our approach involved two components: (i) a one-time calibration experiment that related the PET image space to MRI-visible, non-radioactive location markers within the animal cradle, and (ii) tracking of the location of the cradle markers using MRI. This approach required determining the following three transformations:

- The static affine transform T_P→Cradle_, which maps the PET image coordinate system to a cradle coordinate system defined through the geometric markers in the cradle. This transform needed to be determined only once because the PET scanner was physically locked to the cradle.
- The affine transformation T_Lab→Cradle_, which describes the mapping from the laboratory coordinate system to the cradle coordinate system. The laboratory space is defined as the physical space (in mm) centered at the magnet isocenter and with the z-axis oriented parallel to the magnet axis, x-axis oriented horizontally left-right, and y-axis oriented vertically top-bottom. This transformation must be determined every time the location of the cradle is changed within the magnet, which is generally the case for every experiment where an imaging subject is (re-)positioned on the cradle.
- The affine transformation T_MRI→Lab_, which describes the location of indexed voxels of the MR image in the physical lab space. This transform depends on MR voxel-size, field of view, and the prescription of the imaging slab during MRI acquisition. It is different for every MRI acquisition and can be obtained readily from the headers of the MR images.

Using these definitions, the transformation of PET imaging voxels to the MRI image space is achieved by simple concatenation of the individual transforms and their inverse transforms, respectively:

(Eq. 1) $$x_{MRI}=T_{MRI\to Lab}^{-1}T_{Lab\to \text{Cradle}}^{-1}T_{P\to \text{Cradle}}x_{PET},$$

where ***x***_*PET*_ and ***x***_*MRI*_ are 4-element vectors containing the indices of a single voxel of the PET image and the corresponding coordinates in the MRI image space, respectively, as the first three elements and “1” as the 4^th^ element. The transforms are 4×4 matrices where the top-left 3×3 submatrix is an affine matrix, the 4^th^ column is a 4-element translation vector (with the 4^th^ element being “1”), and all other elements are zero. The PET signal at location ***x***_*MRI*_ in the MRI image is given by:

(Eq. 2) $$P(T_{P\to \text{Cradle}}^{-1}T_{MRI\to Lab}T_{Lab\to \text{Cradle}}x_{MRI}),$$

where P is the array of PET activity values.

**Figure 2.**
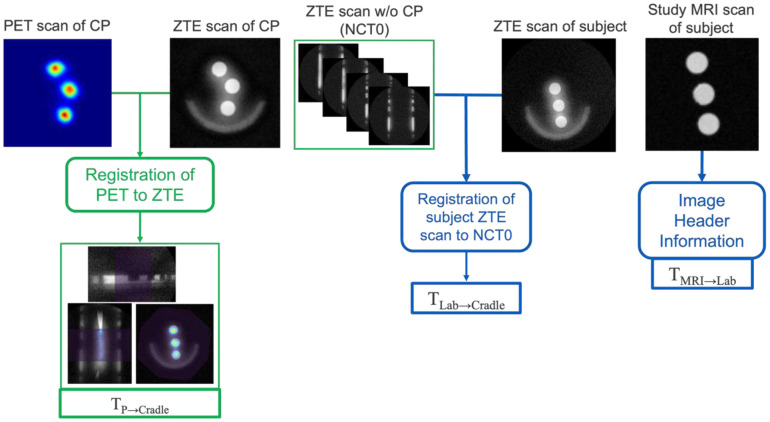
Schematic overview of the steps required to acquire the three affine transformations. The green steps are performed only once while the blue steps must be performed every time a new subject is set up or a new MRI sequence is acquired, respectively.

The conceptual workflow for acquiring the different affine transformations is illustrated schematically in Figure 2 with the green components representing one-time calibration steps and the blue components representing calibration procedures that need to be performed for every experiment.

### Nose cone track (NCT) design

**Figure 3.**
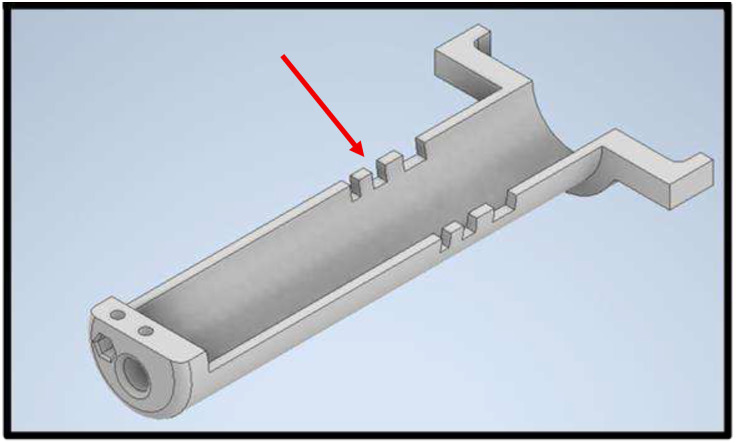
3D model of the modified nose cone track with nock subtraction markers (red arrow).

We replaced the existing NCT with a minimally modified version that featured subtractive nocks serving as geometrical markers to guide image-based registration, illustrated in Figure 3. We manufactured the NCT from acrylonitrile butadiene styrene (ABS) because it is an MR-compatible material (20, 21) that can be imaged using zero-echo time (ZTE) MRI pulse sequences (22–24). The NCT was designed with 3D modelling software (Autodesk Inventor 2025) and 3D printed using fused deposition modelling.

### Determination of the universal PET-to-cradle space transformation (T_P→Cradle_)

**Figure 4.**
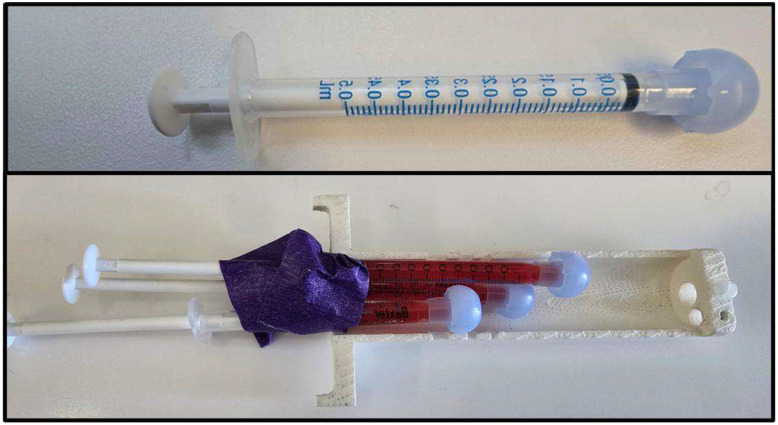
Unfilled 0.5 cc Baxter syringe (top), and three Baxter syringes filled with FDG solution (red dye) on the modified NCT (bottom).

We determined the PET-to-cradle transformation, T_P→Cradle_, by registering simultaneously acquired PET and ZTE-MR images of a dual-modality phantom on the modified NCT. We created the dual-modality phantom by stacking three 0.5 cc Baxter syringes, each filled with 200 μCi of 18F-FDG, as shown in Figure 4. The syringes were aligned in a cascaded way to create a geometry with variations in all three spatial dimensions.

We placed the phantom on the modified NCT and locked the NCT into a fixed position on the cradle assembly, with the MRI volume coil and PET detector installed. We then moved the assembly to the magnet isocenter and acquired incident event data for 10 minutes. At the same time, we acquired ZTE-MRI data with the FOV centered at the magnet isocenter. We used the following parameters for the ZTE pulse sequence: flip angle = 5.172°, (TR) = 2.5 ms, 4 averages, BW = 400 kHz, FOV 50 mm × 50 mm × 50 mm with an isotropic voxel size of 0.1 mm^3^. MRI acquisition time was 35 minutes.

After the acquisition, we pulled the phantom out of the PET-MRI assembly without moving the assembly and repeated the ZTE acquisition (with only the NCT present in the imaging volume). This scan, referred to as “NCT0” in the following, was acquired to serve as a bridge between the universal calibration experiment and the experiment-specific calibration. The absence of the high signal of the dual-modality phantom ensured that the registration algorithm focused entirely on the visible geometric markers.

We used Advanced Normalization Tools (ANTs; Mutual Information metric) (25) for the PET-to-ZTE registration. To precondition the registration, we flipped the PET images manually about the x-axis and initialized the affine transform with a −55 degrees rotation around the z axis, accounting for the approximate orientation of the PET device on the cradle. The resulting affine matrix was T_P→Cradle_.

### Determination of the experiment-specific Laboratory-to-Cradle transformation (T_Lab→Cradle_)

The experiment-specific location of the cradle in the laboratory space was determined through a two-step process. First, a single ZTE calibration scan of the imaging subject was acquired that depicted both the subject and the NCT. Second, the acquired ZTE scan was co-registered with the preexisting NCT0 scan using ridid-body registration (ANTs), yielding T_Lab→Cradle._

The experiment-specific ZTE sequence was optimized for short measurement time: flip angle = 5.172°, (TR) = 2.5 ms, 1 average, BW = 400 kHz, FOV 50 mm × 50 mm × 50 mm with a voxel size of 0.2 mm^3^ (2 minutes, 31 seconds). We considered 2.5 minutes as an acquisition time for a pre-scan that needs to be run once per experiment.

To evaluate the accuracy of the Laboratory-to-Cradle transformation, we performed a systematic experiment in which we successively applied the ZTE sequence to a water phantom. We mimicked different physical locations by adjusting the sequence’s field-of-view (FOV) center locations in every repetition. We then stripped the location header information from the ZTE images and registered them to the NCT0 scan. The change in FOV location was used as a surrogate for the ground truth location change between repetitions. We quantified the accuracy by comparing the location change determined by the registration process with the ground truth change of the FOV center location.

We used a 15 mL deionized water phantom to mimic high signal from tissue on the cradle. We independently varied the FOV center offsets in the x, y, and z directions in increments of 0.05 mm from −0.5 mm to +0.5 mm along each axis. In addition, we used translations across multiple axes using the same step size and range. A total of 90 ZTE scans were acquired.

### Determination of the sequence-specific Laboratory-to-MRI space transformation (sequence-specific T_Lab→MRI_)

The sequence-specific laboratory-to-MRI space transformation, T_Lab→MRI_, was derived directly from the MR image header information without optimization or intensity-driven alignment.

To confirm the correct extraction of T_Lab→MRI_, we imaged the dual-modality phantom with different MRI sequences and voxel geometries and used the extracted T_Lab→MRI_ for transforming the images into the laboratory space. Since the phantom was not moved between scans, all images were expected to align perfectly in laboratory space. We evaluated the alignment using voxel-wise difference images.

A short ZTE calibration scan was acquired to provide a geometry-focused reference, followed by: (i) a 2D turbo RARE T2-weighted scan (TE/TR = 18.46/8563 ms, RARE factor = 4, echo spacing = 20 ms, FOV = 25 × 25 mm^2^, matrix = 250 × 500, slice thickness = 1 mm, 50 slices, bandwidth = 50 kHz, averages =1, acquisition time = 17 min 50 s); and (ii) a 3D FLASH scan (TE/TR = 4.16/12.095 ms, flip angle = 30, FOV = 45 × 45 × 45 mm^2^, matrix = 225 × 225 × 225, isotropic resolution = 0.2 mm, bandwidth = 50 kHz, average =1, acquisition time = 10 min 12 s) acquired at isotropic resolution (0.2 mm). To intentionally perturb the imaging prescription, the RARE and FLASH scans were acquired with prescribed +2 mm offsets in the x, y, and z directions and with varying oblique orientations. Each configuration was repeated twice.

### Application of the complete pipeline

We evaluated the complete pipeline using the dual-modality phantom. To mimic positioning and repositioning of imaging subjects that would result in slightly different physical locations within the magnet, we systematically varied the cradle location within the MR bore.

Before repositioning the cradle, we recorded PET coincidence events for 5 minutes (total activity 600 uCi). Since the location of the phantom within the PET assembly did not change throughout the experiment, these PET images were used for all subsequent experiments.

For the baseline position, the cradle was oriented parallel to the bore axis. The cradle was then moved into the magnet with the center of the PET scanner FOV at the magnet isocenter. For the subsequent experiments, we used the positioning systems digital readout to add location offsets from −2 mm to +2 mm relative to the reference position. We repeated these experiments after angulating the cradle by 5° about the x-axis using the integrated angle adjustment mechanism. These experiments resulted in a total of 9 different cradle positions within the MR scanner. At each position, MRI scans were obtained as described in the previous sections, including the ZTE calibration scan and a 3D FLASH sequence. The FLASH acquisition was prescribed with +2 mm offsets in the x, y, and z directions relative to the ZTE field-of-view (FOV) center. During all angled acquisitions, the FLASH scan prescription was additionally rotated by 6.8° to create an oblique imaging plane relative to the ZTE reference scan. We processed the PET images with the pipeline for each of the cradle positions and MRI scans.

To assess the accuracy of the PET-MRI coregistration, we registered all MRI images to a common space and then applied the resulting transformations to the PET images resulting from application of the pipeline (PET in MR image space). We used one of the FLASH images as a central registration target. Since all images were acquired from the same phantom, we assumed the MRI-based registration to be accurate. We expected that inaccuracies in the PET-to-MRI registration resulting from different cradle positionings would translate to varying locations of the individual PET images in the common FLASH space. To evaluate the accuracy we calculated the voxel-wise standard deviation across all registered PET images, expecting that registration inaccuracies would result in increased variation at the phantom surface.

## Results

### PET-to-cradle transformation

**Figure 5.**
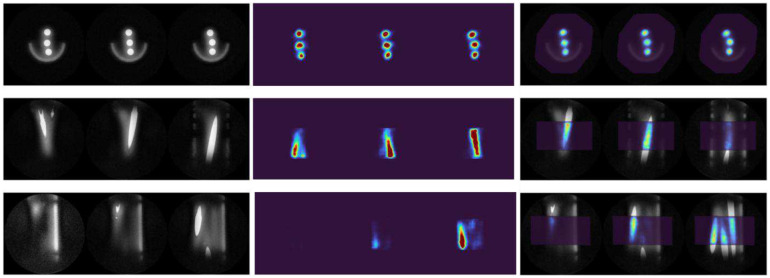
Axial (top), sagittal (middle), and coronal (bottom) slices of the calibration ZTE images (left), the original PET scan (center), and both overlayed (right).

Figure 5 illustrates the ZTE calibration scan, the original raw PET images, and the coregistered images.

**Figure 6.**
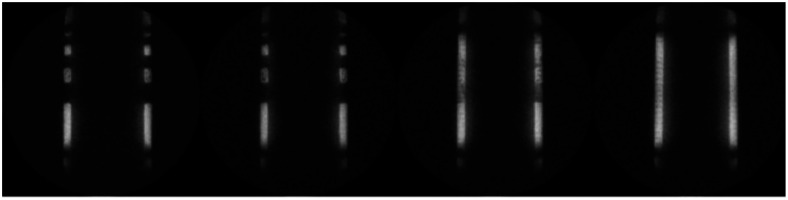
Sagittal views of the NCT0 ZTE scan with the subtractive features clearly identified as breaks in the vertical rails.

Figure 6 illustrates the NCT0 scan with the geometric markers clearly identifiable along the rails and no high signal in the center.

### Cradle-to-MRI transformation

**Figure 7.**
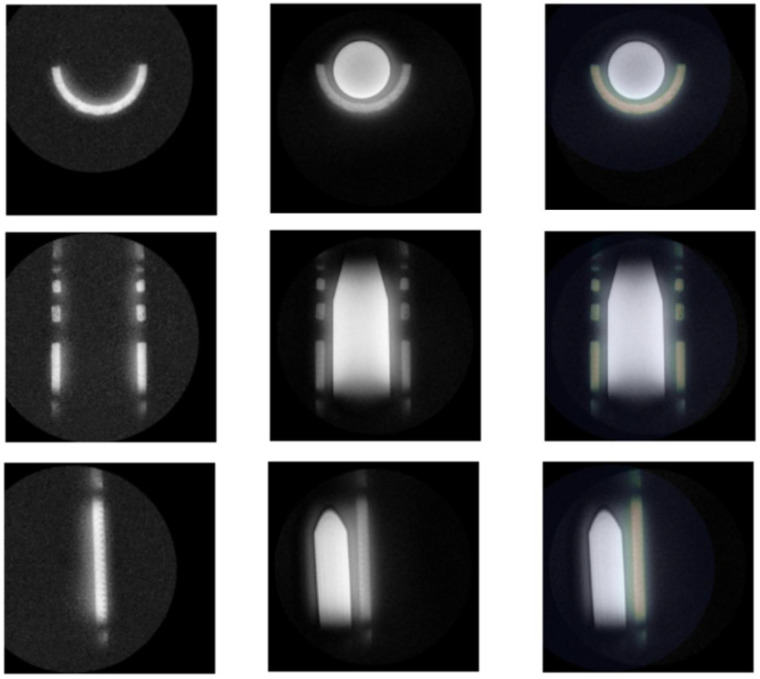
Illustration of the registration of the NCT0 ZTE scan (left) to the water phantom ZTE scan (middle). The right column shows the NCT0 ZTE scan (color) overlaid over the water phantom ZTE scan. Top row are the axial views, middle row sagittal views, and bottom row coronal views.

Figure 7 illustrates the registration of the NCT0 ZTE scan to the experiment-specific water phantom ZTE scan. The registration accuracy in the systematic evaluation was ±0.03 mm, ±0.06 mm, and ±0.06 mm (standard deviation around expected values) in the x, y, and z directions, respectively.

### Application of the Complete Pipeline

**Figure 8.**
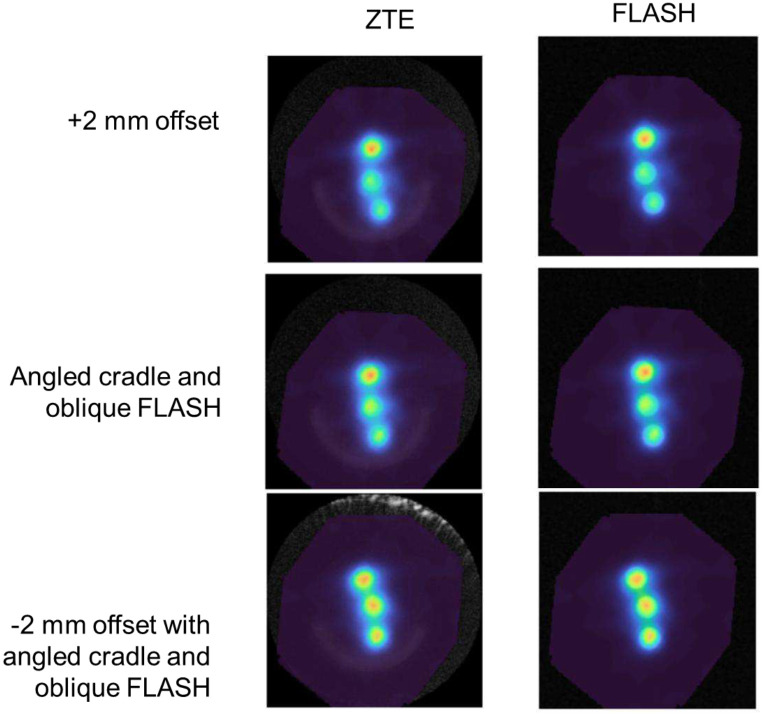
Registered PET images overlaid on the ZTE (left) and FLASH (right) MR images.

Figure 8 illustrates axial views of the PET scan overlaid over the FLASH and ZTE images. The average voxel-based, intensity-normalized (95^th^ percentile) standard deviation across the different MRI sequence acquisitions was 2.6% with a local maximum of 5.4%.

## Discussion

We presented an approach for automated coregistration between simultaneously acquired images generated by a modular MRI-compatible retrofitted PET insert in an ultra-high field MR scanner. The approach provides a new cost-effective method that can be applied and modified for other PET scanners that are not physically fixed to the MRI magnet (26–29). Our approach relied on ZTE-scanning of a 3D printed structure with geometric markers. The ZTE acquisition time of ~2.5 min was sufficiently short to be integrated as an additional prescan in the MRI protocol.

To our knowledge, this represents the first demonstration of applying image-based registration of ZTE scans to automate the coregistration between simultaneously acquired PET and MR scans. While previous studies utilized anatomy-based registration between subject PET and MR scans (8–11), or the use of fiducial markers (30, 31), mutual anatomy is limited in many applications, potentially resulting in registration inaccuracies. Furthermore, small retrofitted PET scanners provide limited physical space to accommodate fiducial markers beside the imaging subject (32).

The Lab-to-Cradle registration was the strongest contributor to registration inaccuracy. However, registration errors remained blow the voxel resolution of the PET images, resulting in limited impact of these inaccuracies on the final coregistration.

A limitation of the study was that the method was only evaluated in phantoms and under controlled conditions. Future work will determine the robustness of the approach in preclinical studies using living animals, where factors such as subject movement and respiration may result in imaging artifacts that could affect the involved registration steps and may require mitigation strategies.

## Conclusion

We developed a novel PET/MR registration method for a non-stationary retrofitted PET detector that relies on MRI-visible ABS plastic imaging markers. Proof of concept experiments in phantoms illustrated the feasibility of the approach.

## Data Availability

The datasets generated and/or analyzed during the current study are not publicly available due to utilizing proprietary 3rd party PET reconstruction software but are available from the corresponding author on reasonable request.

## Availability of data and materials

The datasets generated and/or analysed during the current study are not publicly available due to utilizing proprietary 3^rd^ party PET reconstruction software but are available from the corresponding author on reasonable request.
